# What goes around comes around: Shedding light on today’s doctoral student’s research socialization and who will be the future faculty

**DOI:** 10.1371/journal.pone.0285843

**Published:** 2023-05-25

**Authors:** Sabika Khalid, Gulnar Orynbek, Cai Lianyu, Endale Tadesse

**Affiliations:** 1 College of Teacher Education, Zhejiang Normal University, Jinhua, Zhejiang Province, China; 2 Graduate School of Education, Nazarbayev University, Astana, Kazakhstan; Mayo Clinic Rochester, UNITED STATES

## Abstract

Higher education around the globe is striving to develop rigor and productive doctoral studies that mainly evolve in fostering doctoral students’ research skills by furnishing the necessary socialization process which predicts their future professional and academic decisions. Although scholars investigated the socialization experiences of doctoral students from different perspectives and stages, a large body of evidence is concentrated in western countries that do not define or imply non-western countries like Pakistan. Therefore, the present qualitative study sought to be an icebreaker and stimulant investigation to unfold doctoral students’ socialization experience in research-intensive universities through the lens of Weidman’s socialization framework. After interviewing 24 doctoral students, the findings revealed that doctoral students have high expectations from research universities to enhance their research capabilities. Moreover, most students knew the research’s significance for personal and professional development. The study concluded the progressive and adverse research socialization experiences of doctoral students.

## Introduction

It is well noted that the experience of doctoral students’ socialization process in higher education is more monolithic than specific [1]. Existing literature explains that socialization is a way of knowledge development where individuals adopt the morals, norms, abilities, approaches, and knowledge offered in an institution to develop self-identity [2, 3]. For doctoral students, their self-identity as a researcher is an essential indicator of their professional development. Their academic and research developed during the doctoral study determine their future valuable academic contribution and identities [1, 4], which furnish their career decision to stay in academia or work outside of academia [5–8]. Meanwhile, it is a matter of concern for higher education in the 21st century to obtain an academic with robust research knowledge and skill that can use for next-generation knowledge transfer. It is well known that the decision of doctoral graduates to serve in academics or not determined by what they have become after their doctoral study socialization process [3]. Such arguments are richly discussed in western countries; Thus, the present study intends to shed light on what happens to doctoral students’ research socialization in attempting to develop their professional identities.

## Literature review

### Research socialization in higher education

It became an emerging phenomenon in higher education that cultivating doctoral students for the future professional path are concentrated upon acquiring knowledge, skill, and disposition for their chosen disciplines [9]. The degree and depth of acquisitions that are developed during the doctoral study established and stimulated by their socialization process, which profoundly contributes to the development of the professional identity and the acquisition of substantial knowledge and competency in academic research writing, have become the center of attention for determining the subsequent professional destination of the graduate [3]. Hence, in higher education, doctoral students are expected and ought to socialize in research-related communities intensively by attending academic courses, seminars, and conferences intended to foster their research conceptualizing, writing, data analysis, and a culture of collaborating faculty and peers [10–12]. Unlike undergraduate and postgraduate students, it is apparent that doctoral students’ socialization considerably concentrates on research related to other duties. The coincidence that doctoral students are exhaustively tied up with attending a long series of non-research-related courses could adversely impact the time of research socialization [6]. Instead, doctoral students require a decent interaction with department, faculty members, and peers to shape their knowledge acquisition regarding research which assists in making their future career choices to stay with higher education or work as a professional besides academia [8, 13].

In the meantime, it has been noted that doctoral students are not bounded only to higher education institutions to socialize, rather they are expected to attend international academic conferences, affiliate with professionally related companies, and develop local and international collaboration to develop research networking channels [3, 14, 15]. Socializing with this professional and academic community fosters doctoral students’ self-efficacy in writing and publishing high-quality academic research in the early stages [16, 17]. A growing body of evidence stated that early publications of the doctoral student predict not only their successful academic career but also their supervisors’ research performance and university ranking [e.g., 16, 18, 19]. Hence, university ranking and research culture became the significant contemporary factors that attract doctoral students to recruitment into research programs [20]. Unfortunately, it is a matter of concern for higher education in many developing nations, like Pakistan, where universities possess inadequate research culture, which poses a significant challenge to doctoral student’s research socialization which determines the healthiness of their professional identity development, that leads to incompetent doctoral graduates, and future faculty member.

### Research socialization and doctoral supervision

While discussing the research socialization of doctoral students, it is hard to exclude supervisors who are the "gatekeepers within and outside doctoral study ([1]. Supervisors with strong research backgrounds and competency encourage doctoral students to pursue research as the fundamental area [1, 7], which later promotes their research productivity [21–23]. Moreover, supportive supervisors consider students’ research seriously by giving timely feedback and providing them with research channels that furnish favorable and extended research socialization for the doctoral student [7]. Frequent communication with supervisors builds doctoral students’ research confidence to publish more and obtain more opportunities to collaborate with the scholarly community, which the supervisor advocate (Health, 2002). Given that supervisors have experienced research experts in the field with multiple research collaborations and networks which pass and utilized by the student in pursuit of future professional identity development [15, 23, 24].

On the other hand, literature discussed the reason for inadequate supervision, which revealed that the publish-perish policy in most higher education puts considerable pressure on supervisors and adversely affects communication time with doctoral students [25–28]. Likewise, intentional or unintentional supervisor hand-off approaches of mentorship, which slightly involves only thesis writing, create anxiety and depression among doctoral students, degrading their research socialization motivation and engagement. Thus, to the best of our knowledge, this study is the first study that intended to grasp a comprehensive understanding of doctoral students’ research socialization experiences which is essential to provide robust evidence to forward potential policy and practical implications.

### Research context

Since 2002 after the establishment of the Pakistan Higher Education Commission (HEC), efforts have been made to strengthen the quality of higher education and research to meet the goal of knowledge base economics [18, 29, 30]. Although the recent National Education Policy (2018) indicated that HEC achieved the expansion of higher education in the country, the quality of research in higher education is still a matter of concern [31, 32]. Scholars have argued several factors that impede the research progress in the country and exemplified that one of the factors is the low quality of doctoral students’ research graduates [31, 33, 34]. Keep in view the substantial sensitiveness of doctoral studies to the best of our knowledge; in Pakistan, a dearth of literature tries to unfold doctoral students’ academic and professional identity development [35]. Most studies pinpoint that doctoral graduates from Pakistan higher education join the academic profession without sufficient research skills and competencies that couldn’t be utilized and passed on to doctoral students [e.g., 33]. Hence, due to the low quality of doctoral education in Pakistan, many students are forced to study abroad [32, 36]. Therefore, by taking into the view that the present study is the first of it is kind, the purpose of this study is to explore Pakistani doctoral students’ academic research socialization experiences in universities. Moreover, after an intensive review of national and international literature, the current study sought to achieve the purpose of the study under the following research question, which: -

How do doctoral students experience their research socialization process in research-intensive universities?

### Weidman’s graduate socialization framework

Weidman and colleagues defined socialization as the conscious experience of doctoral students with institution, department, program, faculty, and peers that form personal and professional growth. Socialization supports understanding how doctoral students acquire knowledge and experience in different disciplines [2]. They presented socialization as the holistic process that doctoral students undergo through diverse backgrounds and engagement in research activities such as conferences, workshops, and professional associations [36]. Further, the framework has multi-fact stages of socialization (anticipatory, formal, informal, and personal [2]. The framework considers students’ cognitive outcomes, but knowledge attainment is critical. However, socialization experience differs among individuals. How they interpret, communicate, reflect, and are involved in the event shapes their experiences [37]. Stein and Weidman’s framework provides beyond the formal and informal experiences of doctoral students in the institution [38]. It is conceived as the process that the novice undergoes through the anticipatory stage. They enter the academe with previous skills, values, beliefs, and attitudes.

## Methodology

This qualitative study has adopted a narrative inquiry approach to understand individuals’ experiences and present the ’stories, both the living and telling’ of doctoral students’ socialization in Pakistani higher education, particularly regarding their research socialization process, since “higher education is a reflection of society, and what happens on campus may be no worse than what happens off-campus” [19, 39]. Moreover, this approach views stories or narratives as expressions of meaning in context. A personal narrative is "meaning-making through the shaping of experience; a way of understanding one’s and other’s actions; of organizing events, objects, feelings or thoughts over time (in the past, present and/or future). "Hence, a narrative includes the key elements of lived experience, such as characters, plot, and time [19]. In the present study, our respondent’s narrative concerns are their experience, feeling, how they made sense of them, and what agents and hurdle their research socialization and in what contexts.

### Participants

In this study, participants were selected from two public research-intensive universities in Pakistan, which appeared to be a more suitable option for data collection. Given that these universities have historical aspects of research contribution at the national and international level, possess research-oriented culture with a distinct size of senior academics, and have a long-standing experience of giving out doctoral graduates in various disciplines than other public Pakistan universities. Subsequently, the doctoral students in the study were identified via each university’s doctoral program office (12 from each university) and then invited to participate in our research after obtaining verbal informed consent.

This study was approved by the research ethic committee of Southwest University China (Approval No.082010200000060). To obtain an intended respondent to this research, the study adopted purposive convenient sampling techniques to recruit full-time doctoral students from two research-intensive universities in Pakistan who have at least undergone one year of study. As this study aims to explore doctoral students’ research socialization through the lens of the socialization framework by Weidman et al. [2], recruiting full-time and senior doctoral students was the appropriate action to acquire an extensive understanding of their experience than including part-time and junior doctoral students. Thus, Purposively, students recruited who were full-time enrolled students at these universities and had completed their coursework and comprehensive examination process and are on the way to publications and thesis writing process or those who are done with it and waiting for thesis defense. Eventually, twenty-four doctoral students from different disciplines were selected with a proportional gender ratio who met the study criteria (Table 1). Sample participants were full-time students at these research universities that mainly consisted of three to four years. Most participated either in their second year (n = 11), third year (3), or fourth year (8), and one of participating beyond the fifth year (n = 1) and one in the sixth year (n = 1) of their doctoral programs. These students were considered senior students who had completed their coursework and comprehensive examination and were on the way to conducting research. Most participants were married; only six were single status. (see Table 2).

**Table 1:** Demographic summary of participants.

Respondents	Gender	Marital Status	University	State of Enrollment Year	Disciplines
Respondent 1	M	Married	University A	Third Year	Mathematics
Respondent 2	M	Married	University A	Second Year	Bio-Science
Respondent 3	F	Married	University B	Fourth Year	Economics
Respondent 4	M	Single	University B	Second Year	Management Science
Respondent 5	F	Single	University B	Fourth Year	Mathematics
Respondent 6	F	Married	University A	Fourth Year	Bio-Science
Respondent 7	M	Married	University A	Second Year	Economics
Respondent 8	F	Married	University A	Fourth Year	Electrical Engineering
Respondent 9	F	Married	University B	Third Year	Electrical Engineering
Respondent 10	M	Married	University A	Fourth Year	Electrical Engineering
Respondent 11	F	Single	University B	Fourth Year	Mathematics
Respondent 12	M	Married	University A	Fourth Year	Mathematics
Respondent 13	F	Single	University B	Second Year	Management Science
Respondent 14	F	Married	University B	Second year	Bio-Science
Respondent 15	F	Married	University A	Sixth Year	Economics
Respondent 16	F	Married	University A	Second Year	Economics
Respondent 17	M	Married	University A	Second Year	Management Science
Respondent 18	F	Married	University A	Fourth Year	Physics
Respondent 19	M	Married	University B	Second Year	Electrical Engineering
Respondent 20	F	Single	University B	Second Year	Management Science
Respondent 21	F	Single	University B	Fifth Year	Bio-Science
Respondent 22	M	Married	University A	Second Year	Physics
Respondent 23	M	Married	University B	Second Year	Physics
Respondent 24	M	Married	University B	Third Year	Physics

**Table 2:** Sample interview questions.

Interactive Stages of Socialization			
Stages			Example interview questions
**Anticipatory**	Prospective students (background predispositions)	self-introduction age, status, and job experience	i)Your marital status, if married number of kids, where do you live? ii)What is your major? iii)What you do for living (job); if not working recently what was the previous job experience?
**Formal**	Professional communities, (practitioners, associations)	Research socialization with faculty, supervisor, peers, and academic department	i)What did you get from your doctoral studies until now? (In terms of Research Capacity and skills) ii) How often you meet your supervisor regarding your research work? iii) Explain the research activities your supervisors engaged you in so far? iv)Explain the research activities your supervisors engaged you in so far? v)How does the department support your research learning as a doctoral student (any activities that promote research and interdepartmental activities within and outside your university)? vi)What do you think of your overall progress as compared to your peers?
**Informal**	Professional practitioners,	Research Knowledge, Skill disposition, Personal commitment, and self-identity	Describe your relationship and connection to: i. Faculty members in your and other department regarding research (Conferences, data collection, data analysis work) ii. Your research collaboration with your peers regarding publications iii. Professional associations and collaboration with other Pakistani or International universities regarding research…
**Personal**	Personal Communities	(Family, friends, employers)	i)Has your relationship and connection to these groups changed over time? How? ii)Explain how the university support does specifically, doctoral students to grow and develop a solid research skill? iii)From your doctoral research experience what do your advice for future and current doctoral students?

### Data gathering instrument and process of data collection

Interview questions were designed to answer the study research questions with doctoral students’ research socialization model [2] (see Table 2). Notably, a semi-structured interview strategy allows the researcher to collect detailed accounts of participants’ experiences. Participants were allowed to share their narratives in both languages, English and Urdu; hence, English is a medium of instruction for Pakistan higher education, and researchers have proficiency in both languages, so participants were free to use any language. Interviews were conducted from April-June 2021. Each interview lasted approximately 50 to 60 minutes.

Moreover, mobile phone audio-recording software was used to record interview data.

### Procedure and data analysis

After a professional translational of each interview transcription into English, NVivo 12 software was used to administer a deductive thematic analysis by creating nodes with the sub-nodes under the preconceived themes [39]. The study used a socialization framework; therefore, interpretation-focused coding techniques were adopted to develop codes (See Table 3). This technique allows for exploring and comprehending the experiences of specific settings and environments of doctoral students’ research socialization experiences and using predetermined codes from study research questions under the light of socialization theory to obtain the research objective [39]. This coding approach helps answer the question of what or how [39]. Moreover, the trustworthiness of the data analysis and interpretation was enhanced by consulting with native scholars. We developed codes and themes, checked developing interpretations against alternativeness, and sought divergent cases in the data [19]. Since the data quality depends on the connection between the researchers and participants, we established trust and privacy to ensure quality data collection and appropriate social processes.

**Table 3:** Themes and categories.

Stages	Categories	Themes
**Anticipatory**	Prospective students (background predispositions)	Academic and professional background Doctoral students’ interest in research-intensive universities Admission difficulties Inquiries about the research universities Supervisors’ educational research background and publications Universities research ranking Doctoral students’ Expectations for profession and research growth Research Interest
**Formal**	Professional communities (practitioners, associations)	Research courses Comprehensive examination Research collaboration with peers Research collaboration with the supervisor Supervisor research support and guidance Supervisor concern for thesis writing Supervisors’ research engagement and lack of interest in doctoral students’ research Supervisor’s lack of interest in advanced research
**Informal**	Professional practitioners,	Research collaboration with faculty Departmental research conferences and workshops Funds for research conferences and workshops Research collaboration chances at national and international levels HEC scholarships for research collaboration
**Personal**	Personal Communities	Research confidence Research skills Research knowledge Personal identities Early research publications of doctoral students

### Findings

**Fig 1:**
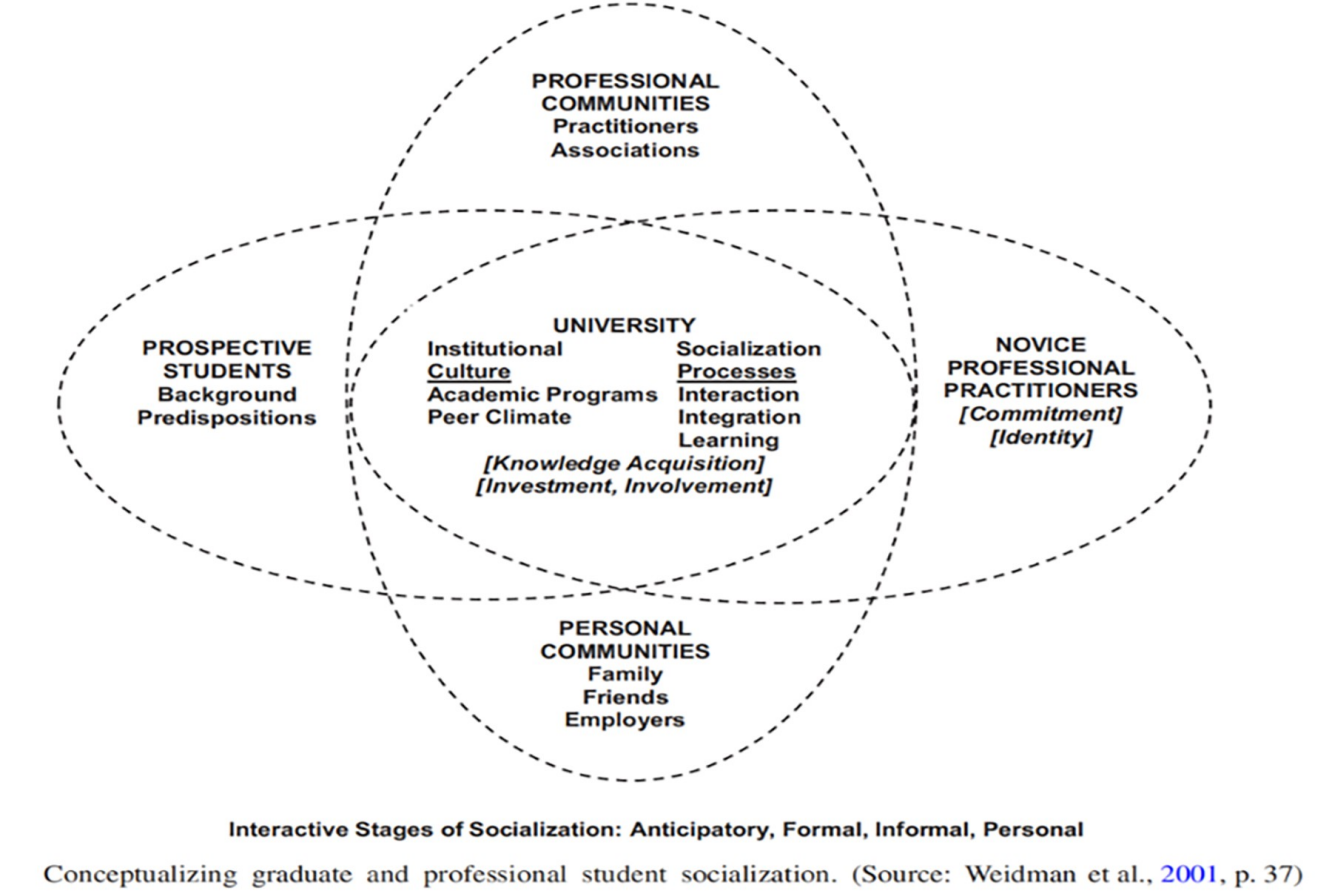
Interactive stages of socialization themselves and the program; secondly, the formal stage interaction with courses, faculty, and peers exposed to institutional culture and practices shapes their understanding; thirdly, the informal stage, the exchange and communication for professional and personal goals boost their self-esteem and confidence, and finally the personal stage of self-identity and change [38], with the core of socialization experience of doctoral students such as knowledge acquisition, investment, and involvement. Throughout socialization, students undergo the knowledge acquisition and transformation stage, which leads from the novices to the professional stage.

Socialization lens support exploring the research socialization experiences of doctoral students (Weidman et al., 2001, p.37) through four interactive stages (anticipatory, formal, informal, and personal) (See Fig 1 and Table 3).

#### Anticipatory stage

In the anticipatory stage, individuals become familiar with a particular organization’s behavioral and attitudinal expectations for their role development. Generally, the process of inquiry and probing information is difficult but essential to know the outcomes of the chosen program [2]. However, in the preliminary narratives of the respondent, they discussed the pulling factors to get started in their doctoral program at these universities. Respondent 1 shared, "university title and research reputation were his pulling factors." Examining the university selections, most respondents discussed the same facts. Respondent 3 remarked, "multiple reasons such as top ranking and *diverse disciplines*, with *competent research-oriented faculties*." In addition, while discussing expectations from the degree, most respondents claimed to become a researcher, such as respondents 2, 19, 21, 23, and 24. "I *want to be a competent researcher and faculty membe*r" (Respondent 2). "Competent researcher who knows how to handle research" (Respondent 19). "I want to be a researcher and want to serve mankind and want to provide my share to fight against the deadly diseases." (Respondent 21)

Few outlined their research-developing concerns. Respondent 7 disclosed, "during my master’s program, I was not satisfied with my research skills, and it worries me because I know how important research is for my professional development … and on the other side, my time and energy".

Many respondents exemplified adding research skills for their professional career growth. *Such as*, *respondent 4 commented*, *"*to enhance my professional development, and to be a *researcher*." Respondent 17 was candid in responding, "I was not interested in research and wanted to obtain the degree for my professional development." Same as Respondent 23, "Want to be a researcher for better job opportunities." Similarly, "researcher defiantly, for my good future" (Respondent 24). One respondent claimed to "acquire robust academic growth and serve society and personal goals" (Respondent 6). While disclosing online information related to programs and faculties, most respondents marked the same respondent 19 informed, "The university has effective websites with ample information regarding the academic and academia".

### Formal stage

The formal stage makes students take the responsibility and privileges appropriate to their past performance and help them to increase maturity [2]. According to the framework, student learning happens during the formal stage through interaction with faculty peers, and traditional courses also play an essential role. Doctoral students explained how they found their academic research environment regarding research courses, conferences, and collaboration within the same department with supervisors and peers and how it influenced their research socialization process. Generally, they shared their research socialization process in general and specific terms. Most spoke about conceptualizing research from departmental structure to research facilities, supervisor research interaction, and peer and faculty research collaboration within the department.

More involvement and engagement in research activities, academic courses, and comprehensive examinations are significant for doctoral students. Considering this, respondent 7 discussed his excitement at learning academic courses, "I point high to university, the quality they teach and the staff they have is competent, the courses were helpful necessary and empowered for my research as compared to the last university." Likewise, respondent 4 expressed excitement: a "pleasant research experience, real research orientation of courses and skills they taught were practical and helpful, and I learned theoretical implications and general introduction within the discipline… everything was systematic." Respondent 9 claimed, "In terms of coursework, I find current university courses more research-oriented than my past one and stimulating in research ideas."

On the other hand, respondent 1 tended to compare courses with their peers "course is not research-oriented more time waste, seminars and workshops are there, but my peers helped me to develop research understanding." Respondent 23 discussed that "competent faculty in research publications; faculty is behind their research publications; not for us… during the coursework they hesitate to share the information with students and few of them undermine students…I believe it is difficult for me to be a strong researcher just after a few general courses." Respondent 6 also emphasized that "strong faculty…, the majority obtained degrees from abroad and had good research understanding in natural sciences and advance research experience with a large number of publications. But for us, limited facilities in the laboratories. Conducting advanced research is an issue for me."

Similarly, almost all respondents encountered the same course experiences and shared an outdated, generic introduction to fields and a lack of research training and skills. While discussing comprehensive examination, respondent 1 indicated, "I cannot entirely agree with the comprehensive extermination system…more time eat up by coursework and comprehensive exams and one and half years for thesis, *this discourages to publish articles*…" Likewise, most other respondents claimed the same fact regarding the argument that bit tricky and a waste of time.

While discussing research communication and interaction with supervisors, respondent 19 declares, "My supervisor is the competent professor, cooperative and available all time for my research help." Similarly, Respondent 10 emphasized that "he is a cooperative and real mentor; he always supports the future researcher and helps his doctoral students. Even at midnight, I emailed him, and he answered me. Even though I think I am lucky enough, he is very cooperative…and sends me different reading literature according to my thesis topic and guides me; he advises me mostly that "more you spread the knowledge, the more you get it. " this is like fragrance spread everywhere." Respondent 16 mentioned that "My supervisor mainly focuses on time. He mostly focuses on meeting deadlines and advises not to think here and there. I think it limits my research and, on the other hand, keeps me on track…well experienced, but the problem is our majors are different, … he referred me to another professor in my field, but I did not get the full assistance." Respondent 24 stated, "*I believe I get successful due to my supervisor’s vast research experience and proper guidance*, *not because of coursework…* I did not get any difficulty. He guided well." While discussing the supervisor’s research communication; the remarks were mixed. Despite this fact, respondent 5 mentioned that "my professor mocked my research ideas and laughed at it, and after the humiliation from him, I gave up doing something exciting for my degree I just follow him".

### Informal stage

The informal stage allows students to develop a solid academic grouping and support system that helps them emotionally, socially, and academically [2].

While discussing the informal research socialization experiences, students discussed their research collaboration with supervisors, peers, and faculties. Considering the supervisors, respondent 1 explained that "my supervisor is a distinguished professor of Pakistan… he is a source of my research linkages with international faculties." Explaining further, respondent 24 concluded, "I am working with other faculty members besides my supervisor; Actually, he is an international degree holder, he is open to research collaboration, which is an unusual practice here…but he discouraged to going for conferences where there are payment self-funded conferences policy rules here." Most students disclosed the similar facts regarding supervisors condemn behavior for research collaboration such as respondent 15 also mentioned similar points that "supervisor discourage the collaboration with faculties and peers just supervisor is considering as Godfather nobody else. It is a strange environment".

While discussing research resources, respondent 21 examined in detail that "our university labs do support my research area… mostly Pakistani faculties try to collaborate with international universities and professors, but they do not bother to coordinate in the same department with their faculties and colleges, which hinders the students’ research…political reasons in the university, they hide their work and do not share it. No linkage between the university and hospitals makes it difficult for students to obtain the research objects, and medical doctors mostly undermine us."

Regarding the collaboration with academic peers, respondents mentioned their diverse aspects and experiences. *respondent 5 discussed* that "peers used to guide me for research, Ph.D., research is all about the networks and collaboration." Contrary to this, respondent 2 pointed out, "*I lack collaboration and interaction with my fellows and peers that is a significant part of natural science students that I feel is missing*…our labs lack collaboration within the same department with faculties and peers…supervisor does not admire it. Secondly, in labs, due to time, we cannot communicate with each other; thirdly, students have different domains and research lines, so it is scarce for us to work with someone new in the field, and it impacts time, and you know supervisors are much concerned about the time and duration of Ph.D., so I do not want to take the risk. Even I know making networks and working on something innovative *is crucial*." Respondent 22 shared some interesting facts "I have competent peers, and we used to attend conferences and seminars and discuss the research with a cup of coffee. However, most surprisingly, peers have good coordination till the conferences and salons; in academic publications, they do not want to share their work; they work as individuals and hide information and knowledge." Similarly, "collaboration with peers is time waste for me." (Respondent 8). While discussing the conferences and seminars, respondent, 11 further elaborated on her research experiences and concluded that, "university supported conferences at the local level, I attended several conferences. However, I went through an odd experience most professors were close to discussing with students and undermined us".

### Personal stage

The final stage of doctoral students’ socialization allows students to reconcile the new image as professionals [2]. At this stage, students start searching for their professional identities and separate themselves from the institution after acquiring knowledge and skills from the department and academic disciplines. Fusion of novice to professional role and self-identity, at this stage student, can reflect on their previous identities and form their professional identity and development [2]. In the light of socialization theory, doctoral students at this stage build their self-identities. Furthermore, they can reflect on themselves as professionals. During the interview, they disclosed their facts about research progression and professional images. Respondent 1 shared that "*research is not the number game*. *It is a stream of understanding knowledge*. *My objective is quality publications; recently*, *I have two Q1(high ranking) journals publications*…with research network… quite confident as a researcher".

While gauging the research development respondent, 12 emphasized, "I have several high impact factor journal publications, and more are in the loop with my supervisors as co-author; I will join academics as a researcher".

Same respondent 20 claimed that "enough publications with high-level international journal with supervisor and peers…research skills are enough to be a researcher. Likewise, respondents 23 and 19 concluded, "Before Ph.D., I was terrified of the word ’Research’ because I did not have the appropriate research skills. However, now I know research does not fear me anymore. Despite that, some respondents shared their lack of research confidence, such as respondent 15’s statement: "I am not a competent researcher. That is why it took me six years to write a thesis with two publications. I want to finish it and want to join the teaching."

## Discussion

The current qualitative study is the first of its kind, which underpins doctoral students’ research socialization experiences. The present section discusses the findings through the lens of the socialization framework, which comprises four interactive stages anticipatory, formal, informal, and personal.

### Anticipatory stage

After a deductive thematic analysis, the finding of the study demonstrates that all participants went through a firm admission process, which explains that participants have rigorous academic profiles and capabilities to endure their research journey with high-ranking universities, which reflects earlier evidence that doctoral students enroll to universities by taking account of their international reputation [16, 20]. Likewise, in this initial socialization stage of doctoral students, the finding indicates that the pulling factors for respondents to participate in doctoral study in these universities were from the research ranking and productive research faculties impressions. Most doctoral students were faculty members in higher education who were aware of research development for their academic and professional growth and wanted to pursue research. Since these doctoral students’ goal to enhance their research capabilities is the result of HEC’s rigorous focus on research and development in the country that makes these doctoral students (faculties) update their research skills to acquire professional development and promotion, the finding is similar with early Pakistani study [19].

Furthermore, the current study finding affirms that almost all the participants are aware of research for the professional and economic development of the country. It is noted that the mindset to acquire professional development by obtaining a doctoral degree was with solid research skills and capacity. However, the insufficient research involvement was the result of family and work obligations which made them acquire the degree with necessary research publications requirements, which contradicts early evidence, which explored that Pakistani doctoral students have less academic research writing knowledge, except writing their graduation thesis to obtain a doctoral degree [40]. Although career progression was the main goal, making these students conduct more research.

Findings revealed that some doctoral students quoted conducting research as a ’pleasant research experience’ demonstrating their confidence and mastery of research skills. Additionally, their pleasant research experience predicts their satisfaction with their research learning process. Since learning research is necessary for doctoral students for their academic and professional careers and new knowledge interpretations [6]. However, the universities research environment contributes to producing new researchers to contribute to knowledge advancement. This study found that most doctoral students compare their research environment (research teaching, research resources, research courses) experience with previous research universities and that current universities are more effective than old ones in research teaching and research resources.

### Formal stage

Based on participants’ word of mouth, the formal stage of socialization occurs through formal research courses, academic seminars, and research-related interaction with the department, faculty, and peers that enable them to acquire research knowledge and capacities [36]. Similarly, a study in the USA showed that doctoral students tended to emphasize more formal processes such as coursework, conferences, and research collaboration with supervisors [3]. Likewise, this study revealed that mostly frequently updates about seminars and salons; the department has the central role in facilitating socialization among doctoral students with peers, and internal and external scholars, which has been mentioned by existing literature that a doctoral student who built robust early research network and collaboration have a promising future in becoming a research productive and competent faculty [16, 17]. However, the finding elucidates that doctoral students couldn’t meet the expectation of exploiting the socialization in attending the research courses since participants mentioned that most courses are inclined to provide general research knowledge or skill than actual and practical comprehension. This finding determines that Pakistani doctoral programs have a hostile socialization learning environment. At the same time, it is stressed by preceding literature that developing feasible curricula for doctoral students that are intended to foster tangible research skills and capacity would ensure the destined socialization it brings [20].

Consequently, in line with previous Pakistani evidence, the finding of this study demonstrates that doctoral students went through publication hardships due to insufficient research idea conceptualization, analyzing, and writing knowledge [19, 30]. In the meantime, the participants in this study addressed that the comprehensive examination taken by the academic professor committee, which every doctoral student has to go through after one year of study, ought to pass considered as the irrelevant element in a formal stage that only brings stress and time waste. Similarly, a large body of literature stated that the long duration of courses and non-research assessments at the doctoral level have an adverse effect on the chance of exposure to research socialization, which leads a doctoral program to be only about taking the course and writing graduation thesis [10–12].

Furthermore, a consecutive volume of literature witnessed supervisors play a great role in directing, supporting, and fostering doctoral students’ formal stage of research socialization. The present study finding shows that doctoral students experience a mixed conception regarding the supervisor’s role in their research socialization. The study noted that most participants title their supervisors’ cooperative,’ ’kind,’ ’supportive,’ ’open-minded to share knowledge,’ ’knowledge channel,’ ’core of research successes,’ and approachable supervisors for their research. Hence, the study supposes that those doctoral students who came across a supervisor that wrought research socialization by involving them in research projects, research collaboration, and funding acquire vital research skills and competency [3]. Nevertheless, the study showed that such a supportive supervisor depends on the supervisor’s origin of the doctoral degree from Pakistan or abroad, supporting emerging evidence [19, 20, 30]. The possible explanation for this finding is that, indeed, those supervisors who obtained their doctoral degree in Pakistani went through research socialization unaccommodating environment.

In contrast, those sent by HCE to western countries’ higher institutions undergo a rigorous doctoral study that abounds in an environment where students socialize interminably. This finding complies with earlier studies stating that by virtue of the endangered quality of doctoral programs, pursuing a doctoral degree abroad has become a recent trend [17, 35]. Alarmingly, it has to be noted that regardless of performance, doctoral graduates from home and abroad will be seen with a different eye in front of HCE during employment and promotion. As a matter of the chain going through the undeveloped and unreformed educational system, faculties obtained from home universities exhibit narrow-minded in collaborating for research with peers and other faculty members and bound their student’s research collaboration and network. In addition, the findings revealed that these supervisors only pay attention to their supervisee thesis writing and neglect other research activities [16]. Simultaneously, a growing body of recent Pakistani studies echoed this finding, which claims that academics who acquired doctoral degrees from overseas universities showed a profound surplus of research than their counterparts who obtained from home universities [19]. It has been well documented that Pakistani faculties are under tremendous pressure from the publish-perish policy of HCE, which is creating considerable ignorance of their doctoral students’ research support.

### Informal stage

Based on the socialization model, in the informal stage, students act and socialize as professionals and less like students to develop their professional identity with faculty and peers [15]. The finding of this study claims that universities are providing opportunities for academic conferences and seminars for research socialization of doctoral students, which allows them to collaborate with explicitly scholarly communities is similar to existing literature [14, 17, 22]. Likewise, it has been pointed out that students who establish a solid association with peers, work colleagues, supervisors, and external scholars experience more competence in research-related tasks, which promotes their professional development and identity. The universities strive to organize internal academic seminars and conferences to facilitate interactions notwithstanding; the finding reveals that due to scarcity of funding and supervisor support, students couldn’t attend external and international educational forums which foster their early research efficacy, collaboration, and network that are essential for their future professional identity [15, 17, 22, 28]. The finding suggests that students nearing the completion of their degrees discuss feeling lost or needing more guidance due to supervisors’ disregard to guide them through the admission to the dissertation writing process. The finding of this study is in accordance with a great deal of evidence which stipulates that academics shows authoritarian and possessive supervising behavior by restricting supervisees from establishing a research collaboration or network with no but with the supervisor for nothing [19, 40]. Therefore, a doctoral student could suffer from considerable anxiety and difficulty in transition in this socialization stage due to unintentional guidance and support [3]. Furthermore, the finding demonstrates that due to the hostile informal setting of socialization, doctoral students avoid discussing and sharing any research information with peers, creating a trend of hidden and sole means of professional development among doctoral students. Faculties supervising behavior is the mirror reflection of their doctoral degree socialization outcome as a supervisee [33].

### Personal stage

Based on the socialization framework, the personal stage is the fusion of novice to professional role and self-identity [2, 3]. Interestingly, the finding indicates that most doctoral students gauge their research through publications and consider the article publication the stage which makes them feel like a researcher, which has been reflected by evidence that certain aspects of the research socialization experiences were perceived as positive contributors to doctoral students’ self-identities as a researcher [6, 14]. While discussing their identities, they felt proud to pronounce their number of publications considered in high-ranked journals as the research publications are a significant part of doctoral students’ academic careers [13]. Additionally, the finding in compliance with the literature asserts that doctoral students’ early publications boost their research efficacy, collaborations, and identity, which help them to obtain high academic careers [8, 16]. On the contrary, doctoral students who cannot build robust research writing skills through the socialization bridge intentionally or intentionally tend to join other professions outside of higher education.

## Conclusion

The purpose of the current study is to explore doctoral students’ research socialization process experience in research-intensive universities through the lens of the socialization framework. The study’s findings suggested that exploring doctoral students’ research socialization experience furnishes an in-depth understanding of the tendency and degree of interaction students experience in the four stages of socialization in the journey of an attempt to develop their professional identity. The study illustrates that students are expected to socialize from the first day simultaneously as doctoral students and professionals. In the meantime, the study concluded that supervisors or faculties play a crucial role in establishing, guiding, and supporting the direction and magnitude of doctoral students’ research socialization process. Moreover, the present study suggested that Pakistani higher education doctoral program needs more compassion to cultivate research-oriented professional than typical doctoral graduates who promise to keep the hostile doctoral program chain.

### Implications and limitations

The study has practical policy implications and recommendations. To develop the research capacities of doctoral students, universities’ leadership and HEC have to provide access to advanced research equipment for natural sciences labs, enabling students to conduct cutting-edge research in the country. Universities administration has to build a strong linkage with industry for the professional research practices of doctoral students. It further revisits the course’s content for advanced methodological and data analysis techniques for natural and social sciences students. In addition, the universities should consider grant writing training workshops and research fellowship funding information and have to reconsider the SMART ways of assessment to evaluate doctoral students instead of an outdated examination system that promotes rote learning.

Alike previous studies, the present study also found that supervisors are more engaged in their research and administrative duties, and less attention and time has been given to doctoral students’ research. Their only focus is on the thesis writing process. HEC and university leadership has to pay attention to doctoral students’ supervision, provide the proper evaluation process that defines supervisors’ and students’ academic relationships, and train faculties to respond to doctoral students’ research mentoring. Furthermore, it might be tremendously significant for doctoral students to provide more scholarships, enabling them to give sufficient time to their research. HEC already has an Oversea and Partial support program for the doctoral student. However, partial scholarships or a loan system for home doctoral students should exist. Possible actions might minimize research development challenges, such as universities creating a research environment by providing academic research groups, building mentoring relationships with professors and peers, and other faculties at the national and international levels. Study findings revealed that supervisors condemned the research collaboration of doctoral students with their academic peers and faculty. Universities’ leadership has to engage doctoral students in collaborative research grants and shared research activities with their peers and other faculty members. Further, the awareness of collaborative research needs to be promoted, and supervisors must avoid these behaviors.

The study has two significant limitations. The first limitation is that this qualitative study used interview data from two research universities in Islamabad, which can only be generalized to the targeted area. Second, the current study could not interview other stakeholders, such as faculty, supervisors, and university leadership, to triangulate doctoral students’ responses. Thus, the present study encourages future qualitative studies to understand the other side of the story, which most studies neglect. Third, the study recruited full-time doctoral students from research-intensive universities; it is recommended that investigations will be conducted to explore part-time and distance learning doctoral students’ research socialization experiences.

## Supporting information

S1 File (RAR)
